# Application of Hyperspectral Imaging in the Assessment of Drought and Salt Stress in Magneto-Primed Triticale Seeds

**DOI:** 10.3390/plants10050835

**Published:** 2021-04-21

**Authors:** Jose Alvarez, Elvira Martinez, Belén Diezma

**Affiliations:** 1 Unidad de Física y Mecánica, ETSIAAB, Universidad Politécnica de Madrid, Av. Puerta de Hierro 2, 28040 Madrid, Spain; elvira.martinez@upm.es; 2 Laboratorio de Propiedades Físicas y Técnicas Avanzadas en Agroalimentación, ETSIAAB, Universidad Politécnica de Madrid, Avda. Puerta de Hierro 2, 28040 Madrid, Spain; belen.diezma@upm.es

**Keywords:** hyperspectral imaging, Triticale, magneto-priming

## Abstract

Hyperspectral imaging is an appropriate method to thoroughly investigate the microscopic structure of internally heterogeneous agro-food products. By using hyperspectral technology, identifying stress symptoms associated with salinity, before a human observer, is possible, and has obvious benefits. The objective of this paper was to prove the suitability of this technique for the analysis of Triticale seeds subjected to both magneto-priming and drought and salt stress conditions, in terms of image differences obtained among treatments. It is known that, on the one hand, drought and salt stress treatments have negative effects on seeds of almost all species, and on the other hand, magneto-priming enhances seed germination parameters. Thus, this study aimed to relate hyperspectral imaging values—neither positive nor negative in themselves—to the effects mentioned above. Two main conclusions were reached: Firstly, the hyperspectral application is a feasible method for exploring the Triticale structure and for making distinctions under different drought and salt stress treatments, in line with the data variability obtained. Secondly, the lower spectral reflectance in some treatments—in the 400–1000 nm segment—is the result of a great number of chemical compounds in the seed that could be related to magneto-priming.

## 1. Introduction

Drought and salinity conditions traditionally affect crop production in arid regions [1]. Nowadays, this problem has also extended into temperate climates. An excessive amount of salts in the soil leads to three types of effects: osmotic, nutritional, and toxic. The osmotic effect can change the natural direction of the water movement from an area containing less salt (soil) to another containing more (plant). Roots absorb less water, making it essential for minerals to remain in the soil (nutritional effect). Finally, the toxic effect is induced by certain ions, such as Na^+^ and Cl^−^ [2,3].

Priming methods are a group of procedures that allow plants to cope more quickly and efficiently with both biotic and abiotic stresses. More specifically, any type of priming produces a greater resistance and a more comprehensive defense response to a given stress in plants. It is not a matter of the direct activation of the defense response, but of a wide range of cellular reactions that were reported to be enhanced by compounds called plant defense inducers [4]. Among the different priming methods, magneto-priming has been selected due to being an efficient one for improving seed germination under drought and salt stress conditions because, among others, it ameliorates the effect of stress by fostering the water uptake by cells [5,6,7,8,9].

Hyperspectral imaging is now emerging as a potential tool for rapid, non-destructive, and automated assessment of foods, plants functional dynamics, soils, etc. It has been proven to be satisfactory when differentiating treatments through the images obtained [10]. Hyperspectral imaging integrates spectroscopic methods and imaging technology. It consists of analyzing the interaction between an incident radiation and a sample, with a view of studying its composition, in such a way that the chemical components present can modify the incident radiation, thus making it very useful for determining the compounds that are present. In the pertinent hyperspectral image, each pixel contains a complete spectrum. No physicochemical analyses are needed, making it ideal for the agri-food sector [11]; for instance, for testing fruit quality [12], obtaining the diagnosis, crop information [13], and predicting moisture content of single corn kernels [14]. By using hyperspectral technology, it is also possible to identify stress symptoms before a human observer does so, which has obvious benefits [15]. One of the most widely used technologies to detect salinity levels in soils is hyperspectral imaging [16], which provides some indexes linking the saline concentration and the reflectance at different wavelengths. It has already been used in dozens of studies, for instance, on the surface of lettuce leaves and to search for the best combination of wavelengths concerning ripening [17].

The technique has also other applications—especially in chemistry and biology—focused on obtaining morphological information of a sample or chemical, and the spatial distribution of its components [18].

The main objective of this paper was to apply the non-destructive, quick, easy-to-use hyperspectral imaging technique to the analysis of Triticale seeds subjected to both magneto-priming and drought and salt stress conditions. There are few studies at the laboratory level that examine changes in the structure or composition of seeds considering both mentioned treatments at a time. Solutions with low water potential were employed in this research to simulate drought and salt stress conditions.

## 2. Materials and Methods

### 2.1. Seeds

Triticale (*X Triticosecale* Wittmack) was one of the first cereal hybrids bred in history (late 19th century), looking like a cross between wheat and rye, but morphologically more similar to the former. It has a great drought tolerance [19], making it ideal for water stress experiments.

Triticale seeds used in this study were provided by The Spanish Office of Vegetable Varieties (Madrid), a public body which ensures homogeneity and quality of seeds.

### 2.2. Magnetic Treatment

A homogeneous and stationary magnetic field was generated in an arrangement of a pair of Helmholtz coils with radius R = 15 cm, switched in parallel and separated by the same distance R. In each coil, the number of turns was 124, and when the current intensity was I = 5 A, the magnetic field strength (B) corresponded to 3.72 mT (millitesla) [20].

**Figure 1:**
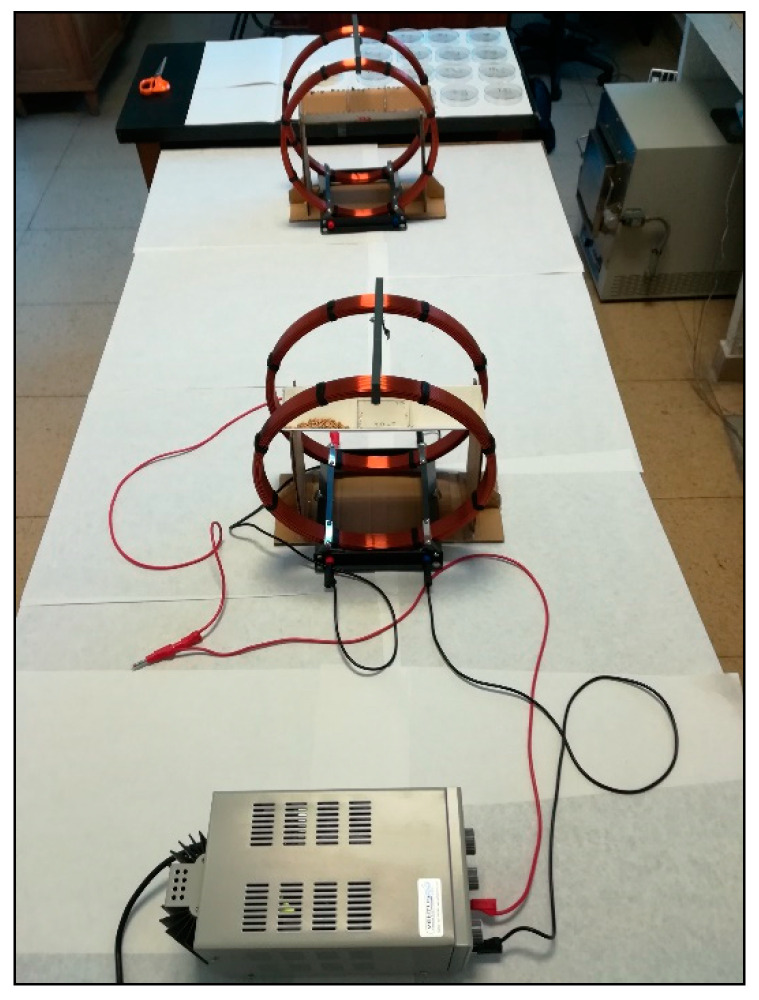
Power supply, cardboard structure and Helmholtz coils for the magnetic treatment. Analogous system not energized for control seeds (background).

Magneto-priming of seeds was performed by putting them in a cardboard structure placed in the axes of coils for 10 h, while non primed seeds were placed in an analogous structure as previously described, but the coils were not energized (Figure 1).

### 2.3. Salt and Drought Stress Generation

Solutions with water potential of Ψ= −1.32 MPa were prepared with PEG6000 (PanReac AppliChem. ITW Reagents, Barcelona, Spain), according to Michel and Kaufmann [21] and Blum [22], and with NaCl following the Van't Hoff equation [23] in order to set drought and salt stress conditions, respectively.

The treatments set up in the experiment are displayed in Table 1. For treatments T3 and T4, 10 mL of distilled water were used, and the time of imbibition in those treatments, and in the ones with solutions (T5–T8), was 5 h.

### 2.4. Hyperspectral Images

**Figure 2:**
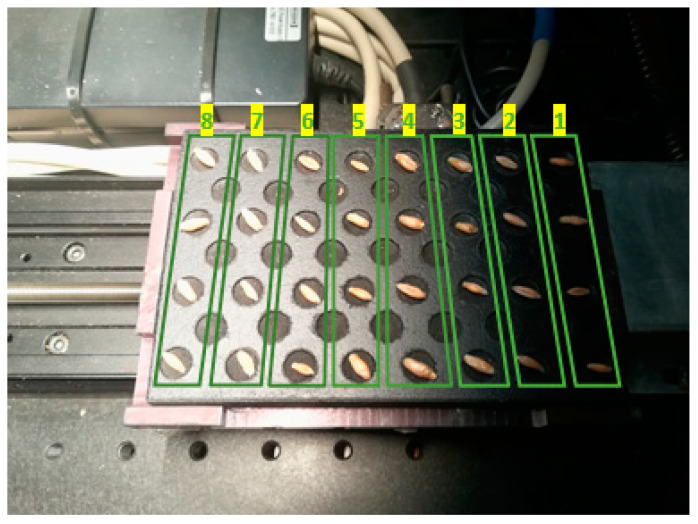
Seed plate inside the system. In the first experiment, seeds were placed on the plate according to their treatment number.

Once seeds were subjected to the treatments described above, they were placed on a 7 × 15 seed plate (Figure 2) in such a way that the first column on the right was filled with control seeds, the adjacent column was left empty, in the third column magneto-primed seeds (T2) were arranged, and so on. In turn, within a column, half of seeds were placed on its ventral—and the other half on its dorsal—side, aiming to study the whole seed surface. An experiment was also conducted in which seeds were placed randomly on the plate in order to eliminate any effects on the results that could accrue from the seed position or the non-uniform light source, also keeping, in this case, the condition of the ventral-dorsal proportion. Once it was confirmed that the seed position on the plate was not influential (there were no differences in the results when seeds were either placed randomly on the plate or arranged by treatment number in columns), two additional experiment replications were carried out with these two seed arrangements.

No apparent symptoms of stress were observed in the external part of the seeds after treatments (Figure 2).

**Figure 3:**
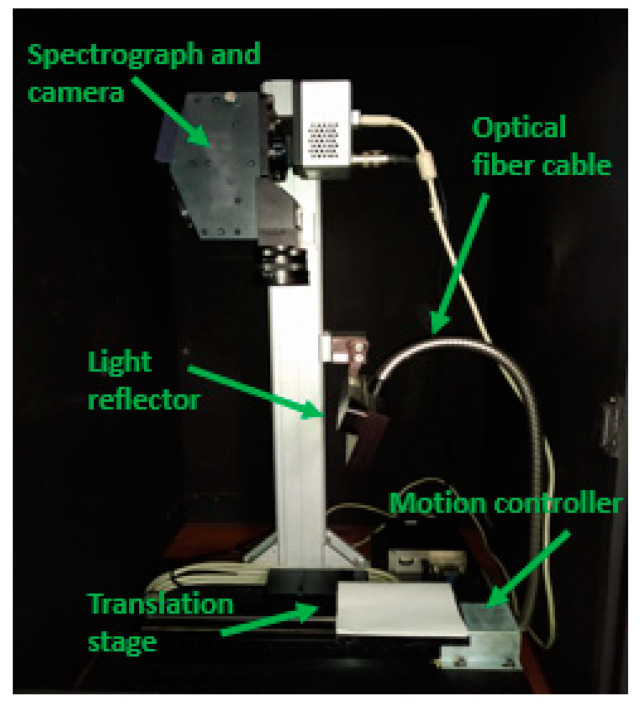
Schematic model of the imaging and analysis setup.

All the devices used to obtain the hyperspectral images were located in the LPF-TAGRALIA laboratory (ETSIAAB, UPM, Madrid, Spain). Hyperspectral images were captured using an EMCCD Luca-R camera (Andor Technology, Belfast, UK) coupled to a VIS-NIR spectrometer (Headwall Photonics Hyperspec-VNIR, Bolton, MA, USA) working in the range of 400–1000 nm as a linear translation stage moving from the right to the left. The mentioned devices were connected to a computer, where image acquisition was possible using Headwall Hyperspec software (Bolton, MA, USA). A schematic model of the total imaging and analysis setup is shown in Figure 3.

Seeds were scanned by covering their entire surface, and data were acquired in two parts of the seed: germ (g) and pericarp (this latter one was named no-germ (*n*) in the present work). The naming pattern used for each seed then had the structure tX_sY_Z, with X being the treatment number (Table 1), Y the repetition number of a treatment (1–4, odd numbers meant seeds placed on their ventral side and even ones on their dorsal side), and Z if the germ or no-germ part of the seed was studied (g/*n*).

Data collected were sufficiently representative since, in every seed, at least 45 spectra were taken in the germ, and 150 in the pericarp, both in horizontal and vertical directions.

Spectral calibration was applied to compensate for anomalous effects coming from the illumination, the detector sensitivity, and the transmission properties of the optics. In spectral calibration, the raw intensity image was corrected with black and white reference images. The black image was acquired with the light source and the camera shutter completely turned off. The white reference image was obtained by imaging a white surface board from spectral on, which has a uniform, stable and high reflectance surface. These two reference images were used to correct the raw images by using the following equation:

(1) $$\mathrm{IR}=\frac{I_{\mathrm{raw}}-{\;I}_{\mathrm{dark}}\;}{I_{\mathrm{white}\;}-{\;I}_{\mathrm{dark}}}$$

where, IR is the calibrated reflectance image, I_raw_ is the raw intensity image measured from the test sample, I_dark_ is the intensity of the black reference, and I_white_ is the white reference intensity.

### 2.5. Data Pre-Processing and Processing

Data were processed using MATLAB (R2020a). In total, approximately 8932 spectra with 189 wavelengths were obtained for each hyperspectral image, which made it necessary to reduce the data dimensionality using the PCA (principal components analysis) procedure. Furthermore, some variables were eliminated to remove the noise.

In the PCA, the greater the number of principal components (PCs), the higher the number of variables composing it, and the lower the amount of information contained. In the fifth component, the explained variance was in the order of 10^−4^, thus the PCs from the fifth one onwards were discarded. As will be pointed out later, the three most excellent PCs were finally selected for a better understanding of the results.

Three-way analysis of variance (ANOVA) was carried out for each PC with the aim of elucidating whether there were significant differences (α of 0.05) between seed arrangements (factor X1), parts (factor X2), or among treatments (factor X3). In the latter case, the ANOVA test results did not mean that there were always differences among all possible treatment combinations, as it was rather a general approach. For this reason, a multiple comparison test was also performed to study the mean differences of all possible combinations within a factor. Finally, the mean spectrum for each treatment (Table 1, factor X3) was generated for reaffirming the results obtained in the PCA, and for studying the wavelength differences between treatment pairs consisting of magneto-primed vs. non-primed seeds: 1 vs. 2, 3 vs. 4, 5 vs. 6, and 7 vs. 8.

## 3. Results

### 3.1. Analysis of Variance

**Figure 4:**
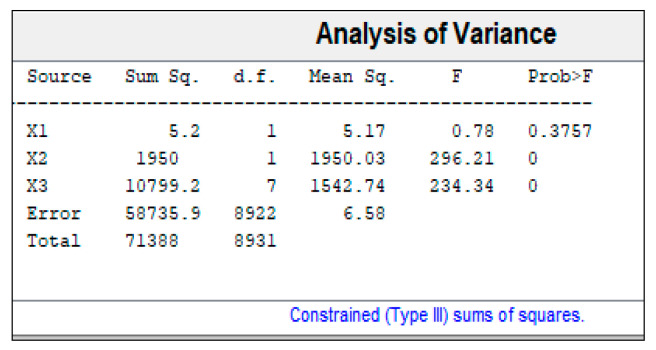
Three-way analysis of variance for PC1 between seed—arrangements (X1), parts (X2) or among treatments (X3). Sum Sq: sum of squares, d.f.: degrees of freedom, Mean Sq.: mean squares for each source, F: F—statistic, Prob: probability.

In light of the results obtained for PC1 (Figure 4), and as Prob > F for the seed position (factor X1) is higher than 0.05, this factor or variable cannot be used to predict germination behaviour. Nevertheless, the seed part (X2) or treatment applied (X3) were similarly decisive for the germination process. Furthermore, it also meant that it was possible to observe the effect of both parameters in the hyperspectral image. Lastly, the fact that the previous two factors were influential, did not mean—in the case of X3—that there were always differences among all possible treatment combinations, instead it was an overview. For this reason, a multiple comparison test was also performed to study the mean differences of all possible treatment combinations concerning X3 and to reaffirm the results obtained for X2.

**Figure 5:**
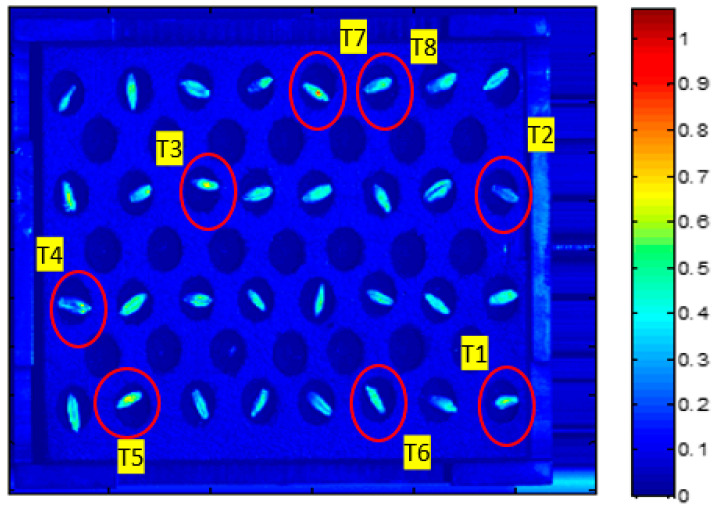
Virtual image computed of plane 614 nm, when seeds were placed randomly. Scale from blue pixels to red pixels (low to high score values). T1 to T8 indicate the treatments’ number to which seeds were subjected (Table 1).

Before covering the next paragraph, it should be noted that when speaking about germination behaviour prediction, the values of parameters from hyperspectral image data are neither positive nor negative in themselves, nevertheless the image (Figure 5) may be effective for detecting differences among the treatments applied. As can be seen in Figure 5, seeds subjected to treatments with magneto-priming (T2, T4, T6 y T8) showed a lower color intensity (especially T2 and T4) than the seeds of the other treatments. Furthermore, treatments containing PEG and NaCl (T4–T8) showed a higher color intensity. These differences may be related to other experiments carried out before [24] where drought and salt stress conditions were found to be harmful for seeds of different species. This will be explained in detail in the discussion section.

### 3.2. Multiple Comparison Test

The results of the multiple comparison test for PC 1, 2, and 5, and factors X2 and X3 are presented below. The authors selected the aforementioned components because of their high suitability for describing the findings obtained, and for discriminating the different variants within a factor.

**Figure 6:**
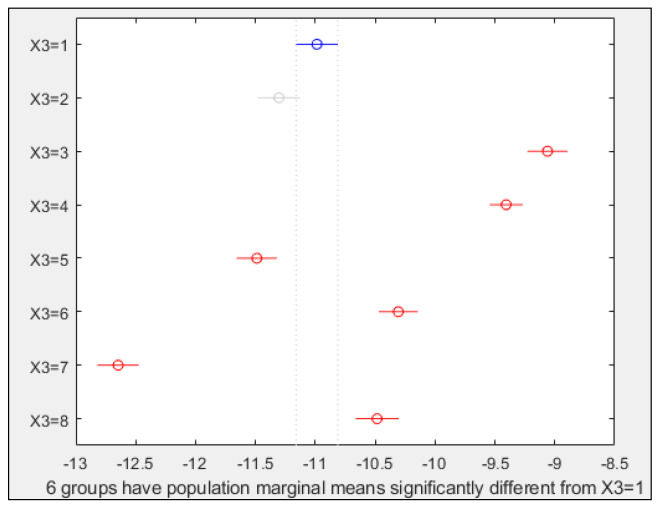
Multiple comparison test for PC1 and factor X3. X axis values correspond to PC1 ones. Y axis numbers correspond to the treatment numbers. Circles represent mean values, and lines the mean confidence intervals. Symbols in red show treatments that were significantly different (α = 0.05) from the control and grey indicates treatments that were not.

**Figure 7:**
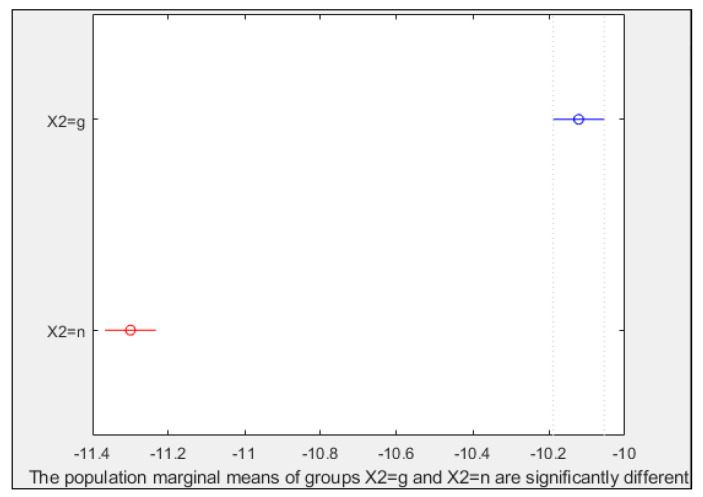
Multiple comparison test for PC1 and factor X2. X axis values correspond to PC1 ones. Y axis letters: g (germ part of the seed), n: pericarp. Circles represent mean values, and lines the mean confidence intervals. Treatments for which the symbols are in red were significantly different (α = 0.05) from the treatment(s) in blue.

Figure 6 shows the results for PC1 and factor X3; in this case, six treatments were significantly different from the control one. T3 and T7 were very different from all the others. Control and T2, or in other words, seeds that are not imbibed in water or any solution, could not be separated statistically, but the other pairs of treatments of non-primed vs. primed seeds could be (T3 vs. T4, T5 vs. T6, and T7 vs. T8). The differences in such pairs increased when the treatment number increased. Thus, in PC1, the treatment-pairs were better separated if water was part of the treatments. It is recalled that Figure 6, and the following ones of this type, averaged the results of germ and no germ parts of the seed. To address this problem, Figure 7 represents the same results of PC1, but this time considering the factor X2 (g/*n*). Seed germ results were significantly different from the pericarp ones. This could also be observed in PC2 and PC5, but the graphs are not shown here.

**Figure 8:**
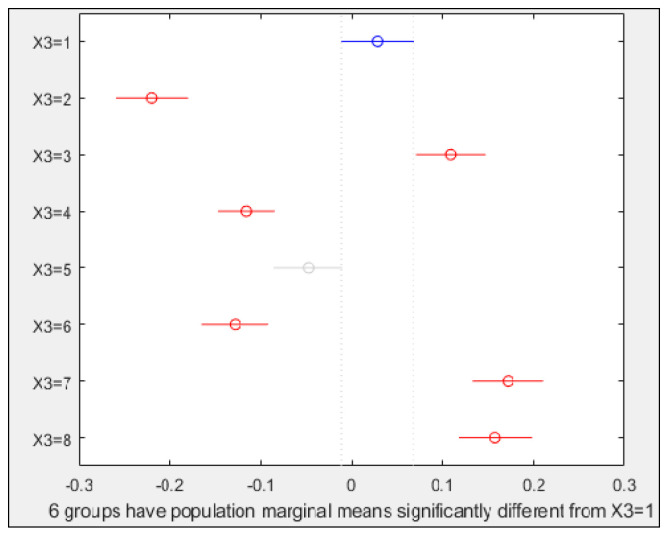
Multiple comparison test for PC2 and factor X3. X axis values correspond to PC2 ones. Y axis numbers correspond to the treatment numbers. Circles represent mean values, and lines the mean confidence intervals. Symbols in red show treatments that were significantly different (α = 0.05) from the control and grey indicates treatments that were not.

Results for PC2 and treatments are shown in Figure 8. All treatments were different from the control, excepting seeds imbibed in the PEG solution (T5). There were always significant differences between pairs of non-primed vs. primed treatments, only T7 and T8 performances were very similar. In contrast to PC1, the difference between pairs for PC2 was always decreasing.

Lastly, PC5 had very similar behavior patterns to PC1, in the sense that the differences between treatment pairs were always increasing, but with the exception that in this case, control (T1) and magneto-primed (T2) seeds could be differentiated.

**Figure 9:**
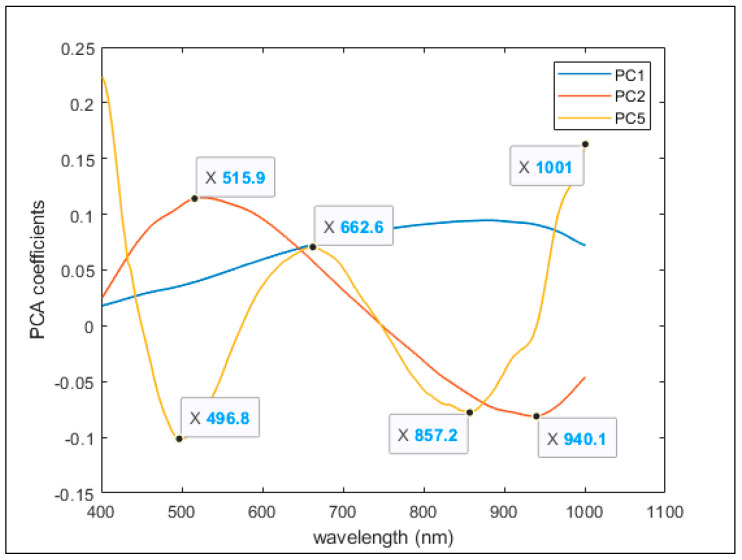
PCA 1, 2 and 5 coefficients at each wavelength and their highest variability.

PC1, PC2 and PC5 coefficients are shown together in Figure 9. In PC2 there were two different regions of the spectrum where the highest variability was located (around 515 and 940 nm). Regarding PC5, the highest variability was observed at wavelengths around 500, 660, 860 and 1000 nm (Figure 9). The variability in PC1 rose when approaching the infrared region of the spectra.

### 3.3. Mean Spectrum

In order to confirm the results obtained in the PCA, the mean spectrum of each treatment was generated. Treatment pair graphs corresponding to magneto-primed vs. non-primed seeds were exceptionally useful in observing the effect of magnetism.

**Figure 10:**
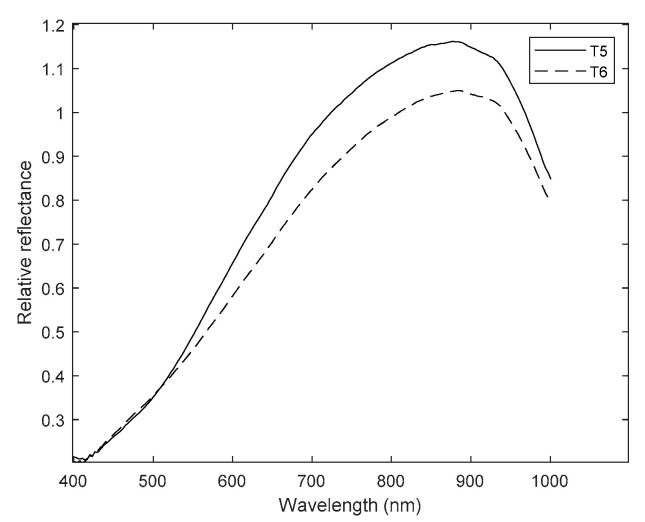
Mean spectra for seeds imbibed in the PEG solution (T5, continuous line) and magneto-primed seeds imbibed in the PEG solution (T6, dashed line). Y axis: relative reflectance and X axis: wavelength (nm).

**Figure 11:**
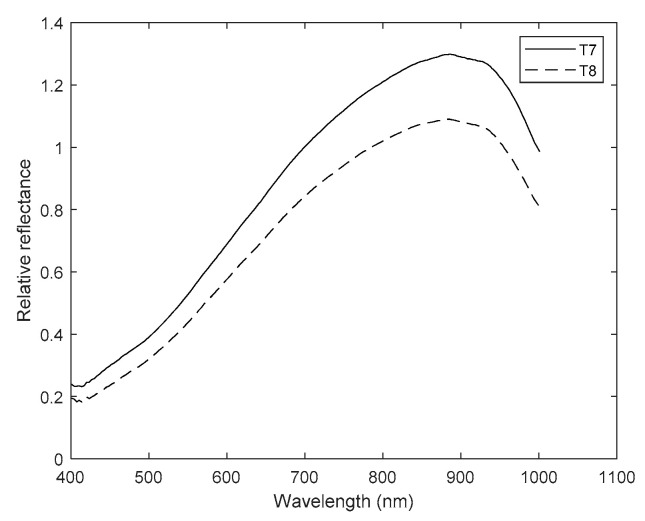
Mean spectra for seeds imbibed in the NaCl solution (T7, continuous line) and magneto-primed seeds imbibed in the NaCl solution (T8, dashed line). Y axis: relative reflectance and X axis: wavelength (nm).

The influence of PC1 and PC5 is noticeable on the graphs, since the differences between pairs of treatments—one with and the other without magnetic treatment—were increasing from T1 vs. T2 up to T7 vs. T8, a pattern already observed in the statistical analysis using the aforementioned components. Only treatments T5 to T8 are shown in Figure 10 and Figure 11.

Obvious spectral differences in Figure 10 and Figure 11 can be appreciated between primed and non-primed seeds in the average spectra of both seed positions, and the germ and pericarp parts. In both figures, the non-primed treatment was above the corresponding primed one almost along the entire spectrum. Therefore, firstly, seeds under drought and salt stress treatments without magnetic induction absorbed less, and thus reflected more light. Secondly, there were fewer chemical compounds in the seeds subjected to such treatments. Only T5 and T6 had a similar trend between 400 and 530 nm approximately, which makes them practically indistinguishable there.

## 4. Discussion

The different seed tonality in Figure 5, may be due to two reasons. The first one is that it could be related to a higher presence of chemical compounds associated with magneto-priming. These compounds may have interacted with the light source of the hyperspectral camera, having resulted in a lower spectral reflectance (i.e., T2 seeds were darker). The second one, as stated in the introduction, may be linked to the fact that magnetism treatments are associated with a higher water uptake, which causes a darker color in seeds subjected to this technique.

PCA led to three principal components useful for different purposes. PC1 is helpful for demonstrating the differences in the hyperspectral images between imbibed seeds subjected to magneto-priming vs. control seeds. These differences could be related to the benefits associated with the magnetism, which was already reported for Triticale by the authors [7,8,20] and supported for other species in other scientific papers [6,24,25]. In all these studies, significant improvements in germination, emergence and water absorption parameters were achieved when using magneto-priming. Moreover, it seems that magneto-priming is more effective when seed species are imbibed in a NaCl solution than in a PEG one [6,24]. This might be consistent with the findings of this paper for PC1 (Figure 6), as magneto-primed seed results were more similar to non-primed ones under PEG (T5 vs. T6) than under NaCl (T7 vs. T8).

Moreover, PC2 is useful for examining the difference in almost all treatment pairs, and for identifying the magneto-priming effect without the need to have recourse to the hydration (since in that PC, the dissimilarity between T1 and T2 was evident). Ultimately, the pre-imbibition of seeds takes up time that can be saved.

In relation to the spectra acquired, it should be stressed that they are consistent with the results of the multiple comparison test made for each PC. It can then be said that seed hyperspectral features are influenced by treatments. Although there are several articles in the literature that used the hyperspectral technique, with a view to make tentative assignments of species possibly present in seeds, it is not easy to find one focused on Triticale.

In general, aggressive treatments, for instance, an ageing one for seeds or other plant parts, generate a higher reflectance in hyperspectral images. Furthermore, light with shorter wavelengths—closer to the visible region—is poorly absorbed when compared to the longer wavelengths nearer the infrared region [26]. These two phenomena were fulfilled in the present study.

Starting with one of the most representative compounds, water has its signal located around 960–970 nm [14] or 934–975 nm [27], depending on that reported by the authors. Most of them agree that, the less in-seed content of water, the more the reflectance achieved [28]. In this experiment treatments including non-primed seeds stored less water because of the spectra higher reflectance. These findings were already reported by Lara et al. [17], except for lettuce leaves subjected to salinity.

The part of the spectrum between 600 and 700 nm is associated with various types of chlorophyll, nevertheless, it is outside the scope of this article because such components are found mainly in leaves. Although it should be highlighted that this is where the carotenoids belonging to the seed pericarp in some species are found [27].

The interval of absorption of organic compounds, such as CH, CH_2_, as well as primary amines or N–H groups, is from 700 nm to 880 nm [14,27].

The strongest peak in every spectrum is around 900 nm. This part of the spectra is one of the most difficult to analyze, given the numerous chemical compounds that reflect in this interval. This region could correspond to the C–H 3rd overtone of starch or cellulose [28] or proteins [14,25,29]. It is clear, indeed, that seeds under treatments with magneto-priming contained a higher amount of the above-mentioned compounds due to the lower reflection observed.

For the above reasons, hyperspectral imaging has become an effective tool to evaluate the chemical composition of seeds [30]. It is also noteworthy for its ability to identify mouldy crop or fruit species faster than the human eye [31], for determining the seed viability [26], and also for detection of infestations [32].

## 5. Conclusions

On the one hand, hyperspectral technology is a feasible method for exploring the Triticale structure and for making distinctions under different drought and salt stress treatments, in line with the hyperspectral data variability obtained. On the other hand, the lower spectral reflectance in some treatments—in the segment between 400–1100 nm—is the result of a great number of chemical compounds in the seed. These differences could arise from the use of magneto-priming since its benefits on germination were already reported in previous studies.

## Figures and Tables

**Table 1:** Treatments of the experiment.

Treatment	Column Number (Figure 2)	Description
T1	1	Control
T2	2	Magneto-primed seeds
T3	3	Soaked in water seeds
T4	4	Soaked in water and magneto-primed seeds
T5	5	Seeds imbibed in the PEG solution
T6	6	Magneto-primed seeds imbibed in the PEG solution
T7	7	Seeds imbibed in the NaCl solution
T8	8	Magneto-primed seeds imbibed in the NaCl solution

## Data Availability

The data presented in this study are available on request from the corresponding author.

## References

[1] Ivorra E. (2015). Desarrollo de Técnicas de Visión Hiperespectral y Tridimensional para el Sector Agroalimentario. Ph.D. Thesis.

[2] Passioura J. (2007). The drought environment: Physical, biological and agricultural perspectives. J. Exp. Bot..

[3] James R.A., Blake C., Byrt C.S., Munns R. (2011). Major genes for Na^+^ exclusion, Nax1 and Nax2 (wheat HKT1; 4 and HKT1; 5), decrease Na+ accumulation in bread wheat leaves under saline and waterlogged conditions. J. Exp. Bot..

[4] Sytar O., Brestic M., Zivcak M., Olsovska K., Kovar M., Shao H., He X. (2017). Applying hyperspectral imaging to explore natural plant diversity towards improving salt stress tolerance. Sci. Total Environ..

[5] Conrath U. (2009). Priming of induced plant defense responses. Adv. Bot. Res..

[6] Kaya M.D., Okçu G., Atak M., Cıkılı Y., Kolsarıcı Ö. (2006). Seed treatments to overcome salt and drought stress during germination in sunflower (*Helianthus annuus* L.). Eur. J. Agron..

[7] Alvarez J., Martinez E., Carbonell V., Florez M. (2020). Effects of polyethylene glycol and sodium chloride stress on water absorption of magneto-primed triticale seeds. Rom. Rep. Phys..

[8] Alvarez J., Martinez E., Florez M., Carbonell V. (2021). Germination Performance and Hydro-Time Model for Magneto-Primed and Osmotic-Stressed Triticale Seeds. Rom. J. Phys..

[9] Sarraf M., Kataria S., Taimourya H., Oliveira Santos L., Menegatti R.D., Jain M., Ihtisham M., Liu S. (2020). Magnetic field (MF) applications in plants: An overview. Plants.

[10] Maffei M.E. (2014). Magnetic field effects on plant growth, development, and evolution. Front. Plant Sci..

[11] Ayala Martini D. (2018). Automatización del Análisis de Imágenes Hiperespectrales para Identificación de Aptitud de Patatas. Bachelor’s Thesis.

[12] Hong T., Li Z., Wu C., Liu M., Qiao J., Wang N. (2007). Review of hyperspectral image technology for non-destructive inspection of fruit quality. Trans. Chin. Soc. Agric. Eng..

[13] Wang J., Nakano K., Ohashi S., Kubota Y., Takizawa K., Sasaki Y. (2011). Detection of external insect infestations in jujube fruit using hyperspectral reflectance imaging. Biosyst. Eng..

[14] Cogdill R.P., Hurburgh C.R., Rippke G.R., Bajic S.J., Jones R.W. (2004). Single-kernel maize analysis by near-infrared hyperspectral imaging. Trans. ASAE.

[15] Lowe A., Harrison N., French A.P. (2017). Hyperspectral image analysis techniques for the detection and classification of the early onset of plant disease and stress. Plant Methods.

[16] Rekha P.N., Gangadharan R., Pillai S.M., Ramanathan G., Panigrahi A. Hyperspectral image processing to detect the soil salinity in coastal watershed. Proceedings of the Fourth International Conference on Advanced Computing (ICoAC).

[17] Lara M.Á., Diezma B., Lleó L., Roger J.M., Garrido Y., Gil M.I., Ruiz-Altisent M. (2016). Hyperspectral imaging to evaluate the effect of irrigation water salinity in lettuce. App. Sci..

[18] Sun D.W. (2010). Hyperspectral Imaging for Food Quality Analysis and Control.

[19] Giunta F., Motzo R., Deidda M. (1993). Effect of drought on yield and yield components of durum wheat and triticale in a Mediterranean environment. Field Crop. Res..

[20] Alvarez J., Carbonell V., Martinez E., Florez M. (2019). The use of Peleg’s equation to model water absorption in triticale (*X Triticosecale* Wittmack) seeds magnetically treated before soaking. Rom. J. Phys..

[21] Michel B.E., Kaufmann M.R. (1973). The osmotic potential of polyethylene glycol 6000. Plant Physiol..

[22] Blum A. Use of PEG to Induce and Control Plant Water Deficit in Experimental Hydroponics’ Culture. Methods. https://plantstress.com/use-of-peg/.

[23] Salisbury F.B., Ross C.W. (1992). Plant Physiology.

[24] Murillo-Amador B., López-Aguilar R., Kaya C., Larrinaga-Mayoral J., Flores-Hernández A. (2002). Comparative effects of NaCl and polyethylene glycol on germination, emergence and seedling growth of cowpea. J. Agron. Crop Sci..

[25] Pietruszewski S., Martínez E. (2015). Magnetic field as a method of improving the quality of sowing material: A review. Int. Agrophysics.

[26] Ambrose A., Kandpal L.M., Kim M.S., Lee W.H., Cho B.K. (2016). High speed measurement of corn seed viability using hyperspectral imaging. Infrared Phys. Technol..

[27] Zhang T., Wei W., Zhao B., Wang R., Li M., Yang L., Wang J., Sun Q. (2018). A reliable methodology for determining seed viability by using hyperspectral data from two sides of wheat seeds. Sensors.

[28] Clevers J.G., Kooistra L., Schaepman M.E. (2010). Estimating canopy water content using hyperspectral remote sensing data. Int. J. Appl. Earth Obs..

[29] Mahesh S., Jayas D.S., Paliwal J., White N.D. (2014). Comparing two statistical discriminant models with a back-propagation neural network model for pairwise classification of location and crop year specific wheat classes at three selected moisture contents using NIR hyperspectral images. Trans. ASABE.

[30] Yang X., Ye Y., Li X., Lau R.Y., Zhang X., Huang X. (2018). Hyperspectral image classification with deep learning models. IEEE Trans. Geosci. Remote.

[31] Chelladurai V., Karuppiah K., Jayas D.S., Fields P.G., White N.D.G. (2014). Detection of *Callosobruchus maculatus* (F.) infestation in soybean using soft X-ray and NIR hyperspectral imaging techniques. J. Stored Prod. Res..

[32] Jiang J., Qiao X., He R. (2016). Use of Near-Infrared hyperspectral images to identify moldy peanuts. J. Food Eng..

